# Supplementary material for: Macrophage Migration Inhibitory Factor (MIF) and D-Dopachrome Tautomerase (DDT): Pathways to Tumorigenesis and Therapeutic Opportunities
Source: Int J Mol Sci. 2024 Apr 29;25(9):4849. doi: 10.3390/ijms25094849 (PMC11084905; doi:10.3390/ijms25094849)
Supplement: Supplementary file 1 [file ijms-25-04849-s001.zip › ijms-2963299-supplementary.pdf]

Supplemental Table S1: A list of cell types expressing MIF and/or DDT as well as their upstream signals and stimuli.

Stimuli	Cell Type	Effect on MIF and/or DDT
Hypoxia, LPS, Reactive Oxygen Species, IFN-Gamma, TNF-alpha, Glucocorticoids[1,2]	Immune Cells (Macrophages; Monocytes, Neutrophils, Eosinophils, Lymphocytes)	Increased MIF and DDT expression and secretion
LPS, CNS Insult (Stroke, Spinal Cord injury, Traumatic Brain Injury)[3-5]	Central and Peripheral Neuronal Cells (Astrocytes, microglia, neuronal cell bodies, oligodendrocytes, Schwann cells)	Increased MIF and DDT expression and secretion
**Glucose[6]**	Pancreatic islet beta cells	Increased MIF secretion
**TNF-alpha[7]**	Insulin-targeted cells (myocytes, cardiomyocytes, adipocytes)	Increased MIF secretion
LPS[8-10]	Hepatocytes	Increased MIF and DDT expression and secretion
Direct Injury[11]	Corneal tissues, Aqueous humor	Increased MIF expression and secretion
Lipopolysaccharide (LPS), UVB[12-15]	Fibroblasts, keratinocytes, Basal layer of epidermis	Increased MIF and DDT expression and secretion
TNF-alpha, LPS[16,17]	Endothelial Cells, Epithelial Cells	Increased MIF Secretion

Supplemental Table S2: A list of MIF and DDT inhibition strategies evaluated in murine cancer models .

Inhibition Strategy	Murine Model	Outcome
Anti-MIF antibody	Prostate Cancer in MF-1 nude mice[18]	- Reduction in tumor growth rate
	B cell Lymphoma in C3H/HeN mice[19]	- Reduction in tumor vascularization
	Esophageal squamous cell carcinoma in BALB/c nude mice[20]	- Reduction in tumor growth rate

		- Reduction in tumor vascularization
	Colorectal Carcinoma in BALB/c mice[21]	- Reduction in tumor mass and hepatic metastases
Anti-DDT antibody	Melanoma in C57Bl/6 mice[1]	- Reduction in tumor growth rate
4-IPP (MIF and DDT inhibitor)	Osteosarcoma in nude mice[22]	- Reduction in tumor burden and metastases<br>- Suppression of osteoclast formation, and induction of osteoblast differentiation
	Bladder Cancer chemically induced by BBN in MIF -/- C57Bl/6 mice[23]	- Reduction in tumor burden, bladder weights, and tumor stages
	Neuroblastoma in athymic mice[24]	- Decreased tumor growth and improved overall survival
4-IPP + anti-CTLA-4,<br>4-IPP + anti-CTLA-4 + anti-PD-L1	Melanoma in C57Bl/6 mice[25]	- Reduction of lung metastases in combination treatments compared to anti-CTLA4 alone<br>- Increased M1 and decreased M2 macrophages in the TME<br>- Increase in CD3+, CD4+, and CD8+ tumor infiltrating lymphocytes in the TME
CPSI-1306 (Chemical Based MIF inhibitor)	Bladder Cancer chemically induced by BBN in C57Bl/6 mice[26]	- Reduction in bladder weight, tumor mass, tumor growth rate, and tumor vasculature
	Triple Negative Breast Cancer in NSG mice[27]	- Reduction in tumor volume and weight<br>- Reduction in proliferation and angiogenic markers
ISO-1 (MIF ~~and DDT~~ inhibitor)	Colorectal Carcinoma in BALB/c mice[21]	- Reduction in tumor growth, weight, vasculature, and hepatic metastases
SCD-19 (MIF inhibitor)	Lung Cancer in C57BL/6 mice[28]	- Reduction in tumor volume
MIF Knockdown	Acute Myeloid Leukemia in lentiviral mediated MIF KD in NSG mice[29]	- Improved overall survival

	Colorectal Carcinoma in siRNA-mediated MIF KD in BALB/c mice[30]	- Reduction in tumor growth<br>- Upregulation of apoptotic markers in tissue<br>- Reduction in hepatic metastases
	shRNA mediated MIF KD of Triple Negative Breast Cancer in NSG mice[27]	- Reduction in tumor volume, weight and pulmonary metastases
	shRNA mediated MIF KD of Breast Cancer cells in BALB/c mice[31]	Increase in tumor-infiltrating CD8+ T cells
MIF and DDT Knockdown	shRNA mediated MIF, DDT, and MIF/DDT KD of Renal Cell Carcinoma transplanted in athymic BALB/c nude mice[32]	- Reduction in tumor vasculature
MIF Knockout	Bladder Cancer chemically induced by BBN in C57Bl/6 mice[33]	- Decrease in tumor vascularization
	Pancreatic Cancer in C57Bl/6 mice[34]	- Reduction in tumor size, weight, and vascularization<br>- Reduction in Tregs, IL-10, and TGF-beta1 in the TME
	MIF KO in MMTV-PyMT (Spontaneous breast cancer) transgenic mice[31]	- Reduction in tumor growth rate and tumor mass
	Pancreatic Cancer in LSL-Kras^{G12D};LSL-Trp53$^{R172H/+}$;Pdx-1-Cre (MKPC) generated mice[35]	- Improvement in overall survival<br>- Reduction in metastasis

BBN: N-butyl-N-(4-hydroxybutyl)-nitrosamine (BBN)

Supplemental Table S3: A list of anti-cancer therapeutics targeting the MIF/DDT/CD74 axis evaluated in Clinical Trials.

Clinical Trials for use in Cancer

Inhibitor	Mechanism	Patient Enrollment	Findings	Study
Imalumab (BAX69)	Recombinant anti-oxMIF antibody	Advanced solid tumors (metastatic colorectal adenocarcinoma, non-small cell lung or ovarian cancer)	- 13/39 (33%) of patients exhibited stable disease<br>- Terminated early due to poor study design, and concerns with safety and efficacy	Phase I NCT01765790

Ibudilast	PDGE2 and allosteric MIF inhibitor	Newly diagnosed and recurrent glioblastoma	- Results not yet published	Phase I/II NCT03782415
Milatuzumab	Anti-CD74 antibody	Relapsed or Refractory Multiple Myeloma	- 5/19 (26%) of patients had stable disease<br>- Moderate reduction of serum B cell levels (median decrease 34%)<br>- No evidence of dose-limiting toxicity<br>- No evidence of rapid drug clearance from the serum	Phase I NCT00421525
		Relapsed or Refractory Chronic Lymphocytic Leukemia	- Overall modest response<br>- Reduction in spleen size, BCL2 levels in malignant cells, and WBC counts	Phase I/II NCT00868478

Supplementary References:

1. Kobold S, Merk M, Hofer L, Peters P, Bucala R, Endres S. The macrophage migration inhibitory factor (MIF)-homologue D-dopachrome tautomerase is a therapeutic target in a murine melanoma model. *Oncotarget*. Jan 15 2014;5(1):103-7. doi:10.18632/oncotarget.1560
2. Lee JP, Foote A, Fan H, et al. Loss of autophagy enhances MIF/macrophage migration inhibitory factor release by macrophages. *Autophagy*. Jun 2 2016;12(6):907-16. doi:10.1080/15548627.2016.1164358
3. Bancroft E, Srinivasan R, Shapiro LA. Macrophage Migration Inhibitory Factor Alters Functional Properties of CA1 Hippocampal Neurons in Mouse Brain Slices. *Int J Mol Sci*. Dec 31 2019;21(1)doi:10.3390/ijms21010276
4. Zhang Y, Yu Z, Ye N, Zhen X. Macrophage migration inhibitory factor (MIF) in CNS diseases: Functional regulation and potential therapeutic indication. *Fundamental Research*. 2023/05/30/ 2023;doi:https://doi.org/10.1016/j.fmre.2023.05.008
5. Hao H, Hou Y, Li A, et al. HIF-1alpha promotes astrocytic production of macrophage migration inhibitory factor following spinal cord injury. *CNS Neurosci Ther*. Dec 2023;29(12):3802-3814. doi:10.1111/cns.14300
6. Waeber G, Calandra T, Roduit R, et al. Insulin secretion is regulated by the glucose-dependent production of islet beta cell macrophage migration inhibitory factor. *Proc Natl Acad Sci U S A*. Apr 29 1997;94(9):4782-7. doi:10.1073/pnas.94.9.4782

7. Sumaiya K, Langford D, Natarajaseenivasan K, Shanmughapriya S. Macrophage migration inhibitory factor (MIF): A multifaceted cytokine regulated by genetic and physiological strategies. *Pharmacol Ther*. May 2022;233:108024. doi:10.1016/j.pharmthera.2021.108024
8. Marin V, Poulsen K, Odena G, et al. Hepatocyte-derived macrophage migration inhibitory factor mediates alcohol-induced liver injury in mice and patients. *J Hepatol*. Nov 2017;67(5):1018-1025. doi:10.1016/j.jhep.2017.06.014
9. Akyildiz M, Gunsar F, Nart D, et al. Macrophage migration inhibitory factor expression and MIF gene -173 G/C polymorphism in nonalcoholic fatty liver disease. *Eur J Gastroenterol Hepatol*. Feb 2010;22(2):192-8. doi:10.1097/MEG.0b013e328331a596
10. Merk M, Zierow S, Leng L, et al. The D-dopachrome tautomerase (DDT) gene product is a cytokine and functional homolog of macrophage migration inhibitory factor (MIF). *Proc Natl Acad Sci U S A*. Aug 23 2011;108(34):E577-85. doi:10.1073/pnas.1102941108
11. Matsuda A, Tagawa Y, Matsuda H, Nishihira J. Expression of macrophage migration inhibitory factor in corneal wound healing in rats. *Invest Ophthalmol Vis Sci*. Jul 1997;38(8):1555-62.
12. Merk M, Mitchell RA, Endres S, Bucala R. D-dopachrome tautomerase (D-DT or MIF-2): doubling the MIF cytokine family. *Cytokine*. Jul 2012;59(1):10-7. doi:10.1016/j.cyto.2012.03.014
13. Abe R, Shimizu T, Ohkawara A, Nishihira J. Enhancement of macrophage migration inhibitory factor (MIF) expression in injured epidermis and cultured fibroblasts. *Biochim Biophys Acta*. Jan 3 2000;1500(1):1-9. doi:10.1016/s0925-4439(99)00080-0
14. Shimizu T, Ohkawara A, Nishihira J, Sakamoto W. Identification of macrophage migration inhibitory factor (MIF) in human skin and its immmunohistochemical localization. *FEBS Lett*. Mar 4 1996;381(3):199-202. doi:10.1016/0014-5793(96)00120-2
15. Yoshihisa Y, Rehman MU, Kondo T, Shimizu T. Role of macrophage migration inhibitory factor in heat-induced apoptosis in keratinocytes. *FASEB J*. Nov 2016;30(11):3870-3877. doi:10.1096/fj.201600408RR
16. Pellowe AS, Sauler M, Hou Y, et al. Endothelial cell-secreted MIF reduces pericyte contractility and enhances neutrophil extravasation. *FASEB J*. Feb 2019;33(2):2171-2186. doi:10.1096/fj.201800480R
17. Hu CT, Guo LL, Feng N, et al. MIF, secreted by human hepatic sinusoidal endothelial cells, promotes chemotaxis and outgrowth of colorectal cancer in liver prometastasis. *Oncotarget*. Sep 8 2015;6(26):22410-23. doi:10.18632/oncotarget.4198
18. Hussain F, Freissmuth M, Volkel D, et al. Human anti-macrophage migration inhibitory factor antibodies inhibit growth of human prostate cancer cells in vitro and in vivo. *Mol Cancer Ther*. Jul 2013;12(7):1223-34. doi:10.1158/1535-7163.MCT-12-0988
19. Chesney J, Metz C, Bacher M, Peng T, Meinhardt A, Bucala R. An essential role for macrophage migration inhibitory factor (MIF) in angiogenesis and the growth of a murine lymphoma. *Mol Med*. Mar 1999;5(3):181-91.
20. Wang XB, Jiang XR, Yu XY, et al. Macrophage inhibitory factor 1 acts as a potential biomarker in patients with esophageal squamous cell carcinoma and is a target for antibody-based therapy. *Cancer Sci*. Feb 2014;105(2):176-85. doi:10.1111/cas.12331
21. He XX, Chen K, Yang J, et al. Macrophage migration inhibitory factor promotes colorectal cancer. *Mol Med*. Jan-Feb 2009;15(1-2):1-10. doi:10.2119/molmed.2008.00107

22. Zheng L, Feng Z, Tao S, et al. Destabilization of macrophage migration inhibitory factor by 4-IPP reduces NF-kappaB/P-TEFb complex-mediated c-Myb transcription to suppress osteosarcoma tumourigenesis. *Clin Transl Med*. Jan 2022;12(1):e652. doi:10.1002/ctm2.652
23. Woolbright BL, Rajendran G, Abbott E, et al. Role of MIF1/MIF2/CD74 interactions in bladder cancer. *J Pathol*. Jan 2023;259(1):46-55. doi:10.1002/path.6018
24. Garcia-Gerique L, Garcia M, Garrido-Garcia A, et al. MIF/CXCR4 signaling axis contributes to survival, invasion, and drug resistance of metastatic neuroblastoma cells in the bone marrow microenvironment. *BMC Cancer*. Jun 17 2022;22(1):669. doi:10.1186/s12885-022-09725-8
25. de Azevedo RA, Shoshan E, Whang S, et al. MIF inhibition as a strategy for overcoming resistance to immune checkpoint blockade therapy in melanoma. *Oncoimmunology*. Dec 6 2020;9(1):1846915. doi:10.1080/2162402X.2020.1846915
26. Choudhary S, Hegde P, Pruitt JR, et al. Macrophage migratory inhibitory factor promotes bladder cancer progression via increasing proliferation and angiogenesis. *Carcinogenesis*. Dec 2013;34(12):2891-9. doi:10.1093/carcin/bgt239
27. Charan M, Das S, Mishra S, et al. Macrophage migration inhibitory factor inhibition as a novel therapeutic approach against triple-negative breast cancer. *Cell Death Dis*. Sep 17 2020;11(9):774. doi:10.1038/s41419-020-02992-y
28. Mawhinney L, Armstrong ME, C OR, et al. Macrophage migration inhibitory factor (MIF) enzymatic activity and lung cancer. *Mol Med*. Apr 16 2015;20(1):729-35. doi:10.2119/molmed.2014.00136
29. Abdul-Aziz AM, Shafat MS, Sun Y, et al. HIF1alpha drives chemokine factor pro-tumoral signaling pathways in acute myeloid leukemia. *Oncogene*. May 2018;37(20):2676-2686. doi:10.1038/s41388-018-0151-1
30. Wu LH, Xia HH, Ma WQ, et al. Macrophage migration inhibitory factor siRNA inhibits hepatic metastases of colorectal cancer cells. *Front Biosci (Landmark Ed)*. Mar 1 2017;22(8):1365-1378. doi:10.2741/4549
31. Balogh KN, Templeton DJ, Cross JV. Macrophage Migration Inhibitory Factor protects cancer cells from immunogenic cell death and impairs anti-tumor immune responses. *PLoS One*. 2018;13(6):e0197702. doi:10.1371/journal.pone.0197702
32. Pasupuleti V, Du W, Gupta Y, et al. Dysregulated D-dopachrome tautomerase, a hypoxia-inducible factor-dependent gene, cooperates with macrophage migration inhibitory factor in renal tumorigenesis. *J Biol Chem*. Feb 7 2014;289(6):3713-23. doi:10.1074/jbc.M113.500694
33. Taylor JA, 3rd, Kuchel GA, Hegde P, et al. Null mutation for macrophage migration inhibitory factor (MIF) is associated with less aggressive bladder cancer in mice. *BMC Cancer*. Jul 24 2007;7:135. doi:10.1186/1471-2407-7-135
34. Suresh V, Dash P, Suklabaidya S, et al. MIF confers survival advantage to pancreatic CAFs by suppressing interferon pathway-induced p53-dependent apoptosis. *FASEB J*. Aug 2022;36(8):e22449. doi:10.1096/fj.202101953R
35. Yang S, He P, Wang J, et al. A Novel MIF Signaling Pathway Drives the Malignant Character of Pancreatic Cancer by Targeting NR3C2. *Cancer Res*. Jul 1 2016;76(13):3838-50. doi:10.1158/0008-5472.CAN-15-2841
